# Dynamic therapeutic response to osimertinib and immunotherapy in an EGFR L747S and L858R co-mutant NSCLC

**DOI:** 10.1093/oncolo/oyag227

**Published:** 2026-06-05

**Authors:** Yuqing Tan, Tengfei Wang, Jing Yu, Wen Ouyang

**Affiliations:** Department of Pulmonary Oncology, Zhongnan Hospital, Wuhan University, Wuhan 430071, China; Department of Pulmonary Oncology, Zhongnan Hospital, Wuhan University, Wuhan 430071, China; Department of Pulmonary Oncology, Zhongnan Hospital, Wuhan University, Wuhan 430071, China; Department of Pulmonary Oncology, Zhongnan Hospital, Wuhan University, Wuhan 430071, China; Hubei Key Laboratory of Tumor Biological Behaviors, Zhongnan Hospital, Wuhan University, Wuhan 430071, China; Hubei Clinical Cancer Study Center, Zhongnan Hospital of Wuhan University, Wuhan 430071, China

**Keywords:** EGFR L747S, lung adenocarcinoma, Osimertinib, Pembrolizumab

## Abstract

**Background:**

Epidermal growth factor receptor-tyrosine kinase inhibitors (EGFR-TKIs) are recommended as the first-line standard treatment for advanced lung adenocarcinoma with common EGFR sensitive mutations (exon 19 E746-A750 deletion and exon 21 L858R). However, the optimal management for rare or compound EGFR mutations remains undefined.

**Case presentation:**

We report a case of advanced lung adenocarcinoma with a rare compound EGFR L747S/L858R mutation. The patient underwent four lines of therapy, showing significant responses to alternating cycles of Osimertinib and Pembrolizumab-chemotherapy, despite intervening disease progression. The clinical course culminated in fatal treatment-induced myelosuppression, with an overall survival exceeding 43 months.

**Conclusion:**

This case underscores dynamic clonal evolution in advanced NSCLC: the initial L747S/L858R clone showed Osimertinib sensitivity, while subsequent resistance revealed a distinct profile (distinct TP53 mutation, MET amplification, high PD-L1/TMB) that explained the durable response to Pembrolizumab. These findings provide crucial evidence for sequential therapy strategies in compound EGFR mutations. Our findings also highlight the utility of Next Generation Sequencing (NGS) in identifying targetable resistance mechanisms, enabling prolonged survival in patients with compound EGFR mutations and offering valuable insights for clinical decision-making.

Key points

- This case demonstrates the real-world clinical efficacy of Osimertinib in advanced NSCLC harboring the rare EGFR L747S/L858R compound mutation, offering a well-tolerated alternative when second-generation TKIs are precluded by tolerability concerns.
- Serial molecular profiling unmasked profound intratumoral heterogeneity, revealing alternating clonal dominance between an EGFR-mutant clone and an EGFR-wildtype subclone under sequential therapeutic pressure.
- The emergent EGFR-wildtype subclone was characterized by a unique molecular profile: high-level MET amplification, high TMB, a novel TP53 driver mutation, and strong PD-L1 expression, which drived a robust and durable response to immunotherapy.
- These results emphasize that dynamic re-biopsy is essential for understanding radiological “mixed responses” and tailoring sequential therapies to specific clones.”

## Introduction

Non-small cell lung cancer (NSCLC) is the most common subtype of lung cancer, with epidermal growth factor receptor (EGFR) mutations serving as key oncogenic drivers. The two predominant EGFR mutations—exon 19 deletion mutation and the exon 21 L858R point mutation—account for approximately 80% of all EGFR-mutant NSCLC cases. Among the rare mutations, the EGFR L747S mutation represents an uncommon but clinically relevant variant, characterized by a missense substitution of serine for leucine at codon 747 within exon 19.^1^ Several reports have indicated EGFR L747S mutation is resistant to first-generation EGFR-TKIs but might be responsive to afatinib,^2–5^ with limited cases also suggesting sensitivity to Osimertinib.^4–7^ However, the treatment of this rare mutation is challenged by two key uncertainties: the unconfirmed efficacy of frontline Osimertinib, and the undefined optimal strategy after resistance develops. To address these uncertainties, we report a case of NSCLC harboring a compound EGFR L747S/L858R mutation. The longitudinal results confirmed both the efficacy of Osimertinib against this mutation and the activity of subsequent Pembrolizumab-based chemotherapy after EGFR-TKI resistance. Our case may inform therapeutic strategies for patients with comparable rare mutations.

## Case story

**Figure 1.**
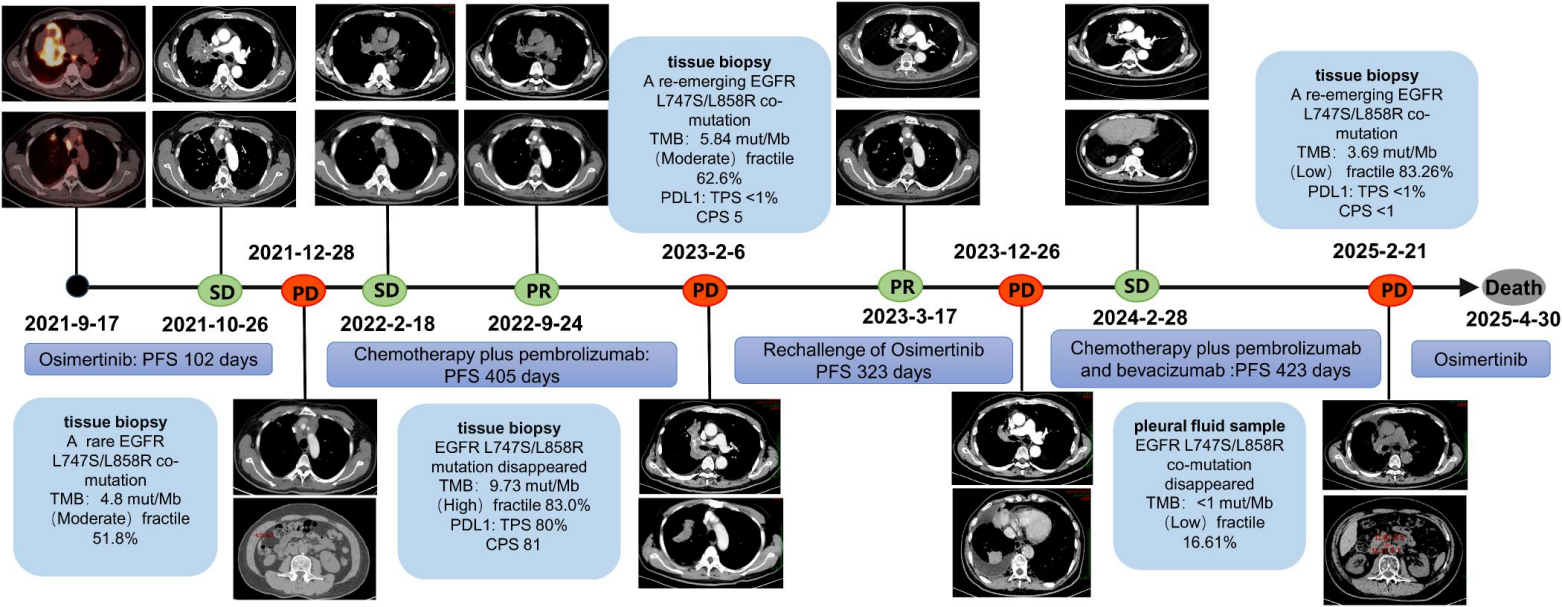
Time line of clinical history for a lung cancer patient with a rare EGFR L747S and L858R co-mutation. CPS, combined positive score; PD, progressive disease; PD-L1, programmed death-ligand 1; PFS, progression-free survival; PR, partial response; SD, stable disease; TMB, tumor mutational burden; TPS, tumor proportion score.

In September 2021, a 68-year-old man with a smoking history of over 20 years underwent a fludeoxyglucose F 18 positron emission tomography (PET) scan for cough and progressive dyspnea; the scan revealed a mass in the upper lobe of the right lung (Figure 1). The patient was staged as cT4N3M1a, IVA, according to the eighth TNM classification after a comprehensive examination. A biopsy of the right supraclavicular lymph node identified poorly differentiated adenocarcinoma originating from lung. Molecular testing was performed using the Next Generation Sequencing (NGS) platform (Table 1), which showed compound mutations: exon 19 L747S and exon 21 L858R EGFR.

**Table 1.** The variant allelic frequency results of five Next Generation Sequencing (NGS).

	First		Second		Third		Fourth		Fifth	
	Plasma	Tissue	Plasma	Tissue	Plasma	Tissue	Plasma	Tissue	Plasma	Tissue
**EGFR L747S**	0	9.60%	0	0	NA	13.62%	NA	0	NA	19.51%
**EGFR L858R**	0.20%	15.60%	0	0	NA	28.14%	NA	0	NA	22.60%
**TP53**	0.50%	22.40%	0.71%	25.90%	NA	57.14%	NA	0	NA	29.20%
**Type**	c.574C>T (p.Q192*)		p.R249S		c.574C>T (p.Q192*)		0		c.574C>T (p.Q192*)	
**TMB (muts/Mb)**	3.80	4.80	7.80	9.73	NA	5.84	NA	<1	NA	3.69
**Fractile**	60.10%	51.80%	81.53%	83.02%	NA	62.63%	NA	16.61%	NA	83.26%
**PD-L1**	NA	NA	NA	80%	NA	<1%	NA	0	NA	<1%
**MET**	0	0	NA	25.27%	NA	0	NA	0	NA	0

Abbreviations: EGFR, epidermal growth factor receptor; MET, mesenchymal-epithelial transition factor; NA, not available; PD-L1, programmed death-ligand 1; TMB, tumor mutational burden.

Given the potential resistance conferred by the L747S mutation to first-generation EGFR-TKIs,^4^^,^^5^ and the adverse effects associated with afatinib,^8^ the patient started on orally 80 mg Osimertinib daily in September 2021. The first follow-up CT scan was performed after 1 month of treatment, which displayed stable disease (SD) with evident tumor shrinkage according to the Response Evaluation Criteria in Solid Tumors, version 1.1(RECIST v1.1). A rapid regression of cough and dyspnea was observed, and the patient became totally asymptomatic after 2 months of Osimertinib therapy.

Then after 3 months of Osimertinib treatment, the patient’s cough worsened again in December 2021, and a subsequent CT scan revealed the emergence of a new peritoneal nodule and enlarged mediastinal lymph nodes, despite continued shrinkage of the primary right lung mass. Although the primary tumor responded, the development of new lesions defined this as progressive disease (PD), representing a classic mixed response. A CT scan-guided re-biopsy of the enlarged anterior mediastinal lymph nodes confirmed lung adenocarcinoma again. NGS of the re-biopsy tissue revealed the disappearance of the baseline L858R and L747S mutations, accompanied by the emergence of a de novo TP53 mutation (p.R249S) that replaced the original p. Q192 variant. Concurrently, MET amplification was detected. Furthermore, the tumor exhibited high tumor mutational burden (TMB) and high PD-L1 expression (Table 1). Subsequently, the patient started Pembrolizumab with PP (Pemetrexed plus carboplatin) chemotherapy on January 8, 2022. Due to intolerance of nausea and vomiting, the regimen was switched to single-agent Pemetrexed plus Pembrolizumab on January 29, 2022. After 3 cycles of Pembrolizumab-based treatment, the patient’s cough was relieved again, the peritoneal nodule disappeared, the lung mass continued to shrink, and the mediastinal lymph nodes were significantly smaller, which was confirmed as PR after 10 cycles of Pembrolizumab. Unfortunately, the patient complained of progressive shortness of breath after activities in February 2023.

### Molecular tumor board

A CT scan showed PD with an enlarged mass in the upper lobe of the right lung and a small pleural effusion. CT-guided re-biopsy and NGS revealed reemergence of L747S (c.2240T>C, VAF: 13.62%) and L858R (c.2573T>G, VAF: 28.14%) mutations, with a moderate TMB of 5.84 muts/Mb. The previously detected MET mutation (c.1433A>G, VAF: 25.27%) was no longer detectable, and the original TP53 p. Q192 mutation returned, replacing the TP53 p. R249S mutation from the second biopsy. In this case, MET amplification represents a primary oncogenic driver of a distinct tumor subclone (EGFR-wildtype, unique TP53 mutation), not a bypass mechanism to overcome EGFR-TKI inhibition. Accordingly, progression was driven by clonal switching: suppression of the initial EGFR-mutant subclone and outgrowth of an EGFR-wildtype, MET-amplified subclone—distinct from classical acquired MET amplification. After immunotherapy suppressed this subclone, the original EGFR-mutant clone reemerged, restoring Osimertinib sensitivity and supporting rechallenge.^9^

### Patient update

The patient rechallenged Osimertinib orally 80 mg daily in March 2023. The CT scan after 1 month of treatment indicated PR, and the patient became asymptomatic following 2 months of therapy. Osimertinib was continued.

Until December 2023, the patient experienced recurrent cough. A CT scan revealed PD with an enlarged mass in the right lobe mass and a concomitant increase in right pleural effusion. NGS results showed the re-disappearance of L858R and L747S mutations. Treatment was subsequently switched to Pemetrexed plus Bevacizumab and Pembrolizumab. After three cycles, the patient became totally asymptomatic, achieving SD in February 2024. However, after 10 months of Pembrolizumab-based chemoimmunotherapy, dyspnea recurred in February 2025. Follow-up imaging confirmed PD in the lungs and retroperitoneal lymph nodes. Histopathological analysis of a CT-guided biopsy confirmed metastatic lung adenocarcinoma, and NGS of the enlarged mass revealed reemergence of the compound EGFR L747S/L858R mutation (Table 1). Following one month of Osimertinib rechallenge, a follow-up scan showed a mixed response. Consequently, chemotherapy with Pemetrexed (900 mg) in combination with Osimertinib was administered in March 2025. Unfortunately, the patient developed an infection secondary to treatment-related myelosuppression and succumbed on April 30, 2025.

## Discussion

The management of NSCLC harboring the classical EGFR L858R mutation is well-established.^8^ However, the presence of the rare L747S mutation complicates this paradigm.^4–5^ L747S is classified as a PACC (P-loop and αC-helix compressing) mutation, for which preclinical studies have demonstrated greater selectivity of second-generation EGFR-TKIs.^10^ However, our patient harbored a compound L858R/L747S mutation, comprising both classical-like and PACC mutants. Currently, no consensus exists regarding which mutation dominates treatment sensitivity in compound genotypes. Moreover, the predictive value of PACC classification established in single-mutant models remains to be validated in this setting. Beyond molecular considerations, emerging evidence from case reports and prospective series provides some support for Osimertinib. Grolleau et al.^6^ reported the case with L747S mutation treated by Osimertinib, experienced up to 12 months duration. Similarly, a prospective study of 36 patients with uncommon EGFR mutations reported a median PFS of 8.2 months with Osimertinib therapy,^11^ supporting its clinical activity against select rare variants. Comparison of safety data from the ARCHER 1050 and FLAURA trials demonstrates that Osimertinib is associated with significantly lower rates of grade ≥3 gastrointestinal and dermatologic adverse events than Dacomitinib.^12^^,^^13^ The treatment decision was made in 2021, when NCCN guidelines had established Osimertinib as the preferred first-line therapy for advanced EGFR-mutant NSCLC.^14^ Based on contemporaneous guideline recommendations, its favorable safety profile, and the lack of prospective data for second-generation TKIs in compound mutations, the patient was initiated on Osimertinib as the initial strategy. Consistent with above observations, our patient achieved SD with evident tumor shrinkage on Osimertinib, confirming initial drug sensitivity. However, PD occurred after only three months, coinciding with the disappearance of the L747S and L858R mutations. This temporal association suggests that loss of the Osimertinib-sensitive L747S clone may represent a novel mechanism of acquired resistance.

There is currently no standardized treatment for patients developing resistance to EGFR-TKIs, particularly those with rare compound mutations. Post-resistance options include chemotherapy combined with immune checkpoint inhibitors (ICIs) and/or anti-angiogenic agents,^15^^,^^16^ as well as targeted therapies against emergent drivers, including MET, HER2, or HER3.^17^ Tumors harboring MET amplification often display an immunosuppressive phenotype characterized by reduced CD8+ T cell infiltration, enrichment of immunosuppressive cells, and upregulation of immune checkpoint molecules such as PD-L1.^18^ Paradoxically, MET-amplified tumors also exhibit higher TMB compared to MET ex14-mutant tumors, and frequently co-occur with TP53 mutations.^19^ High TMB can generate abundant neoantigens, potentially enhancing immunogenicity and susceptibility to ICIs. Moreover, TP53 mutations promote genomic instability and are associated with increased TMB and PD-L1 expression in lung adenocarcinoma.^20^ Thus, the convergence of MET amplification, high TMB, TP53 mutation, and PD-L1 positivity may create a unique molecular context where the immunosuppressive effects of MET are counterbalanced by enhanced immunogenicity, leading to improved responses to immunotherapy.^21^ These molecular features have direct clinical implications. The role of ICIs in EGFR-mutant NSCLC has evolved, with pembrolizumab currently approved in this setting.^22^ Trials, including ORIENT-31 and IMpower150, have demonstrated that chemoimmunotherapy with or without bevacizumab might improve outcomes over chemotherapy alone in EGFR-mutant NSCLC after TKI resistance.^23^^,^^24^ In our case, the patient achieved a significant and durable response to pembrolizumab plus chemotherapy lasting 23 months. This exceptional outcome may be attributed to a convergence of favorable molecular features following Osimertinib resistance: emergence of a distinct subclone with MET amplification accompanied by high TMB (9.73 muts/Mb), a concurrent TP53 mutation, strong PD-L1 expression (TPS 80%), and the absence of the original EGFR L858R and L747S mutations during this period.

Notably, after the patient developed resistance to pembrolizumab-based chemotherapy, the lost L858R and L747S mutations reappeared upon re-biopsy. EGFR-TKI rechallenge following initial progression has been previously reported as a potentially effective strategy.^9^ As expected, when L858R and L747S mutations returned, Osimertinib rechallenge elicited a PR. To investigate the clonal dynamics underlying this complex treatment trajectory, we performed serial molecular profiling across five sequential biopsies. This analysis revealed a complex but coherent pattern of clonal evolution (Table 1). The first, third, and fifth biopsies shared an identical TP53 mutation (c.574C>T, p. Q192*), providing definitive evidence of a common clonal origin. The second biopsy harbored a distinct TP53 mutation (p.R249S), reflecting intratumoral heterogeneity. It is well established that TP53 mutations can exist as clonal driver alterations in one tumor region while presenting as subclonal alterations in others.^25^ We acknowledge the inherent limitation of single-region biopsy sampling, which cannot definitively exclude the possibility of a synchronous or metachronous second primary tumor. However, the presence of an identical TP53 mutation across three temporally distinct biopsies (first, third, and fifth) strongly supports a shared clonal origin. The distinct TP53 mutation observed in the second biopsy is more plausibly explained by branching clonal evolution under therapeutic pressure rather than by a completely independent tumor.

## Conclusion

In summary, this case highlights the complexity of tumor evolution in advanced non-small cell lung cancer under sequential therapeutic pressure. The disease course reflects dynamic shifts in clonal dominance among genetically distinct tumor populations rather than simple linear resistance. The initial EGFR L747S/L858R-mutant clone showed sustained sensitivity to Osimertinib, whereas a genetically distinct clone characterized by TP53 mutation, strong PD-L1 expression, high TMB, and MET amplification subsequently emerged and drove disease progression. These molecular features may explain the favorable response observed with subsequent pembrolizumab-based chemotherapy. This case underscores the importance of repeated molecular profiling to capture evolving clonal dynamics and guide adaptive treatment strategies in advanced NSCLC. While the findings strongly suggest profound intratumoral heterogeneity, the possibility of a synchronous or metachronous second primary tumor cannot be completely excluded.

## Data Availability

The data supporting the findings of this study are available from the corresponding author upon reasonable request. However, specific patient medical records containing identifiable private information are not publicly available due to privacy and ethical restrictions.

## References

[1] Nie L , Wang Y-N, Hsu J-M, et al Nuclear export signal mutation of epidermal growth factor receptor enhances malignant phenotypes of cancer cells. Am J Cancer Res. 2023;13:1209-1239. 37168336 PMC10164793

[2] Liang S-K , Ko J-C, Yang JC-H, et al Afatinib is effective in the treatment of lung adenocarcinoma with uncommon EGFR p.L747P and p.L747S mutations. Lung Cancer. 2019;133:103-109. 10.1016/j.lungcan.2019.05.019 31200815

[3] He Q , Shi X, Zhu H, et al A case treated with crizotinib after secondary MET amplification of A double rare L747S and G719S EGFR mutation pulmonary sarcomatoid carcinoma. Ann Oncol. 2020;31:544-546. 10.1016/j.annonc.2020.01.010 32122695

[4] Chiba M , Togashi Y, Bannno E, et al Efficacy of irreversible EGFR-TKIs for the uncommon secondary resistant EGFR mutations L747S, D761Y, and T854A. BMC Cancer. 2017;17:281. 10.1186/s12885-017-3263-z 28424065 PMC5395747

[5] Huang Y , Pang L, Lao J, et al Rare mutations at EGFR L747 position: molecular characteristics and superior response to afatinib in NSCLC patients. ESMO Open. 2025;10:105558. 10.1016/j.esmoop.2025.105558 40834500 PMC12529304

[6] Grolleau E , Haddad V, Boissière L, et al Clinical efficacy of osimertinib in a patient presenting a double EGFR L747S and G719C mutation. J Thorac Oncol. 2019;14:e151-e153. 10.1016/j.jtho.2019.02.034 31235042

[7] Luo YW , Lin L, Shufeng C, et al Osimertinib treatment response in a patient with lung adenocarcinoma harboring two rare EGFR mutations: a case report. Oncol Lett. 2024;28:501. ARTN50110.3892/ol.2024.14634 39233826 10.3892/ol.2024.14634 PMC11369848

[8] Park K , Wan-Teck Lim D, Okamoto I, et al First-line afatinib for the treatment of EGFR mutation-positive non-small-cell lung cancer in the ‘real-world’ clinical setting. Ther Adv Med Oncol. 2019;11:1758835919836374. 10.1177/1758835919836374 31019567 PMC6466470

[9] Metro G , Bonaiti A, Birocchi I, et al Tracking and tackling the tumor dynamics clonal evolution: osimertinib rechallenge after interval therapy might be an effective treatment approach in epidermal growth factor receptor (EGFR)-mutant non-small cell lung cancer (NSCLC). J Thorac Dis. 2022;14:816-819. 10.21037/jtd-22-98 35572898 PMC9096286

[10] Robichaux JP , Le X, Vijayan RSK, et al Structure-based classification predicts drug response in EGFR-mutant NSCLC. Nature. 2021;597:732-737. 10.1038/s41586-021-03898-1 34526717 PMC8481125

[11] Cho JH , Lim SH, An HJ, et al Osimertinib for patients with non–Small-Cell lung cancer harboring uncommon EGFR mutations: a multicenter, Open-Label, phase II trial (KCSG-LU15-09). JCO. 2020;38:488-495. 10.1200/jco.19.00931 PMC7098834 31825714

[12] Wu Y-L , Cheng Y, Zhou X, et al Dacomitinib versus gefitinib as first-line treatment for patients with EGFR-mutation-positive non-small-cell lung cancer (ARCHER 1050): a randomised, open-label, phase 3 trial. Lancet Oncol. 2017;18:1454-1466. 10.1016/s1470-2045(17)30608-3 28958502

[13] Soria J-C , Ohe Y, Vansteenkiste J, et al Osimertinib in untreated EGFR-Mutated advanced non–small-cell lung cancer. N Engl J Med. 2018;378:113-125. 10.1056/NEJMoa1713137 29151359

[14] Ettinger DS , Wood DE, Aisner DL, et al NCCN guidelines insights: non–small cell lung cancer, version 2.2021. J Natl Compr Canc Netw. 2021;19:254-266. 10.6004/jnccn.2021.0013 33668021

[15] Garcia J , Hurwitz HI, Sandler AB, et al Bevacizumab (avastin^®^) in cancer treatment: a review of 15 years of clinical experience and future outlook. Cancer Treat Rev. 2020;86:102017. 10.1016/j.ctrv.2020.102017 32335505

[16] Dhabhar B , Sahoo TP, Akshay JK. Role of immunotherapy in metastatic EGFRm NSCLC. Indian J Cancer. 2022;59:S68-S79. 10.4103/ijc.IJC_49_21 35343192

[17] Dong R-F , Zhu M-L, Liu M-M, et al EGFR mutation mediates resistance to EGFR tyrosine kinase inhibitors in NSCLC: from molecular mechanisms to clinical research. Pharmacol Res. 2021;167:105583. 10.1016/j.phrs.2021.105583 33775864

[18] Ye L , Wang W, Li H, et al Targeting the MET gene: unveiling therapeutic opportunities in immunotherapy within the tumor immune microenvironment of non-small cell lung cancer. Ther Adv Med Oncol. 2024;16:17588359241290733. 10.1177/17588359241290733 39483139 PMC11526239

[19] Kron A , Scheffler M, Heydt C, et al Genetic heterogeneity of MET-Aberrant NSCLC and its impact on the outcome of immunotherapy. J Thorac Oncol. 2021;16:572-582. 10.1016/j.jtho.2020.11.017 33309988

[20] Dong Z-Y , Zhang C, Li Y-F, et al Genetic and immune profiles of solid predominant lung adenocarcinoma reveal potential immunotherapeutic strategies. J Thorac Oncol. 2018;13:85-96. 10.1016/j.jtho.2017.10.020 29127022

[21] Liu M , Minne RL, Javeri S, et al Immune and genomic heterogeneity of MET-Altered non–small cell lung cancer. JCO Precis Oncol. 2025;9:e2500048. 10.1200/po-25-00048 41004700

[22] Abdel-Rahman O. Evaluation of efficacy and safety of different pembrolizumab dose/schedules in treatment of non-small-cell lung cancer and melanoma: a systematic review. Immunotherapy. 2016;8:1383-1391. 10.2217/imt-2016-0075 27892744

[23] Lu S , Wu L, Jian H, et al Sintilimab plus bevacizumab biosimilar IBI305 and chemotherapy for patients with EGFR-mutated non-squamous non-small-cell lung cancer who progressed on EGFR tyrosine-kinase inhibitor therapy (ORIENT-31): first interim results from a randomised, double-blind, multicentre, phase 3 trial. Lancet Oncol. 2022;23:1167-1179. 10.1016/s1470-2045(22)00382-5 35908558

[24] Lam TC , Tsang KC, Choi HC, et al Combination atezolizumab, bevacizumab, pemetrexed and carboplatin for metastatic EGFR mutated NSCLC after TKI failure. Lung Cancer. 2021;159:18-26. 10.1016/j.lungcan.2021.07.004 34303276

[25] Jamal-Hanjani M , Wilson GA, McGranahan N, et al; TRACERx Consortium. Tracking the evolution of Non-Small-Cell lung cancer. N Engl J Med. 2017;376:2109-2121. 10.1056/NEJMoa1616288 Epub 2017/04/27. 28445112

